# Mechanochemical Synergism of Reactive Oxygen Species Influences on RBC Membrane

**DOI:** 10.3390/ijms24065952

**Published:** 2023-03-21

**Authors:** Elena Kozlova, Viktoria Sergunova, Ekaterina Sherstyukova, Andrey Grechko, Snezhanna Lyapunova, Vladimir Inozemtsev, Aleksandr Kozlov, Olga Gudkova, Aleksandr Chernysh

**Affiliations:** 1 Laboratory of Biophysics of Cell Membranes under Critical State, Federal Research and Clinical Center of Intensive Care Medicine and Rehabilitology, V.A. Negovsky Research Institute of General Reanimatology, 107031 Moscow, Russia; 2 Department of Medical and Biological Physics, Sechenov First Moscow State Medical University (Sechenov University), 119991 Moscow, Russia; 3 Faculty of Physics, Federal State Budget Educational Institution of Higher Education M.V. Lomonosov Moscow State University (Lomonosov MSU), 119234 Moscow, Russia; 4 Administration, Federal Research and Clinical Center of Intensive Care Medicine and Rehabilitology, 107031 Moscow, Russia

**Keywords:** RBC membranes, ROS, mechanochemical synergism, kinetic model, hydroxyl radical, hydrogen peroxide, Fenton reaction

## Abstract

The influences of various factors on blood lead to the formation of extra reactive oxygen species (ROS), resulting in the disruption of morphology and functions of red blood cells (RBCs). This study considers the mechanisms of the mechanochemical synergism of $OH^{•}$ free radicals, which are most active in the initiation of lipid peroxidation (LPO) in RBC membranes, and $H_{2}O_{2}$ molecules, the largest typical diffusion path. Using kinetic models of differential equations describing $C_{H_{2}O_{2}}\left(t\right)\;$ and $C_{OH^{•}}\left(t\right)$, we discuss two levels of mechanochemical synergism that occur simultaneously: (1) synergism that ensures the delivery of highly active free radicals $OH^{•}$ to RBC membranes and (2) a positive feedback system between $H_{2}O_{2}$ and $OH^{•}$, resulting in the partial restoration of spent molecules. As a result of these ROS synergisms, the efficiency of LPO in RBC membranes sharply increases. In blood, the appearance of $OH^{•}$ free radicals is due to the interaction of $H_{2}O_{2}$ molecules with free iron ions ($Fe^{2+}$) which arise as a result of heme degradation. We experimentally established the quantitative dependences of $C_{OH^{•}\;}\left(C_{H_{2}O_{2}}\right)$ using the methods of spectrophotometry and nonlinear curve fitting. This study extends the analysis of the influence of ROS mechanisms in RBC suspensions.

## 1. Introduction

The most important processes in living organisms are redox processes, which regulate the state and morphology of red blood cell (RBC) membranes. The combination of oxidative and antioxidant processes helps to maintain metabolic processes in RBCs, tissues, and the body as a whole [1,2].

A wide range of reactive oxygen species (ROS) in aqueous solutions plays a major role in oxidative processes in RBCs. Some examples of active oxygen-containing compounds are $\;OH^{•}$, $O_{2}^{•-}$, $HO_{2}^{•}$, $H_{2}O_{2}$, $OH^{+}$, $OH^{-}$, $O_{2},\;O_{2}^{1},\;\;\mathrm{and}\;HO_{2}^{-}$ [3,4,5].

ROS are formed under the action of various factors in the organism, in particular, in blood, such as toxins, radiation, etc. [6,7,8]. ROS production can also occur in various diseases [9,10,11] as a result of bacteria and viruses [12,13,14], such as COVID-19 [15,16], and during the long-term storage of packed red blood cells (pRBCs) [17,18,19]. Oxidative stress is frequently described as an imbalance between the production of reactive oxygen species in the biological system and their ability to defend through sophisticated antioxidant machinery [20,21]. Oxidant molecules act on the cell membrane, resulting in the distortion of RBC homeostasis. A major source of oxidative stress in blood is extracellular hemoglobin (Hb) originating from the hemolysis of RBCs [22]. Free $\;Fe^{2+}$ arising from heme degradation is an active component of the Fenton reaction, which plays an important role in redox processes in the blood [22,23].

The consequence of oxidative stress development is the initiation of lipid peroxidation (LPO), which is a chain process of destructive reactions in RBC membranes. This leads to the formation of LPO products, in particular, hydroperoxides in RBC membrane lipids [4,24]. The most important product in LPO initiation is free radical $OH^{•}$. Biophysical consequences of LPO in biological membranes include the formation of bonds between atoms with a large difference in their electronegativity and the consequent local appearance of polar covalent bonds between atoms in the tails of membrane lipids [25]. This is the biophysical basis for the formation of local dipoles in lipid tails, which leads to a change in the permeability of water molecules and ions through the biological membrane [24,25]. Thus, the passive transport of ions through the RBC membrane changes, and osmotic phenomena can occur. The development of oxidative processes also leads to the disturbance of protein structures and their aggregation [26]. As a result, the effects of ROS on membrane lipids and proteins contribute to the violation of RBC cell morphology. The surface membrane nanostructure and cytoskeleton configuration can also change [27]. Biological systems also use ROS to damage bacteria and viruses.

The appearance of ROS and their interaction with biological objects is a multistage process. For instance, the influence of ionizing radiation on biological objects can be broken down into four consecutive stages [4]: physical, physicochemical, chemical, and biological stages. Their peculiarity is a very strong difference in the characteristic duration of each stage, from 10^−13^ s to several minutes or even years. Other influences in the first stage include a weakening of the immune system during various diseases, resulting in the initiation of other stages of development [15,28,29,30]. Multiple stages must be taken into account in the biophysical and mathematical analysis of the effects of ROS on biological membranes.

The mutual transformation of ROS during various chemical reactions is a distinctive feature. Kinetic models such as the Malthus, Verhulst, and Lotka–Volterra models are usually used for the mathematical modeling of the change in the number of specimens during biological and chemical processes [31,32,33]. In the case of mutual interactions of ROS in biological systems, the complex spatiotemporal pattern must be taken into account. At the same time, the activities of ROS, as well as their typical lifetimes and diffusion paths, differ significantly. Analysis of the influence of ROS must also consider their wide range of influences on biological objects, in particular, the cell membrane. To study oxidative stress, RBCs are often used as a research model [34,35]. Moreover, such analyses must take into account that the most active ROS that influences lipids in RBC membranes is radical $OH^{•}$ [36].

At different levels of organization of a biological system, the synergism of the action of factors plays an important role [37,38,39,40]. This is especially important for the blood system.

In this work, based on a biophysical approach and a mathematical model, we established synergism between ROS in RBC suspension. The effectiveness of the action of several ROS on RBCs in terms of their synergism is greater than their separate actions. Among ROS, we singled out those that demonstrate the most pronounced synergistic effect in RBC suspensions, leading to a significant biological effect.

The biophysical approach and mathematical modelling of ROS synergism can expand our understanding of the oxidative influence on the membrane structure and morphology of RBCs, as well as their function.

## 2. Results and Discussion

### 2.1. ROS in Blood Quantitative Study of $H_{2}O_{2}$ to $OH^{•}$ Conversion Due to the Fenton Reaction in In Vitro Experiment

**Figure 1:**
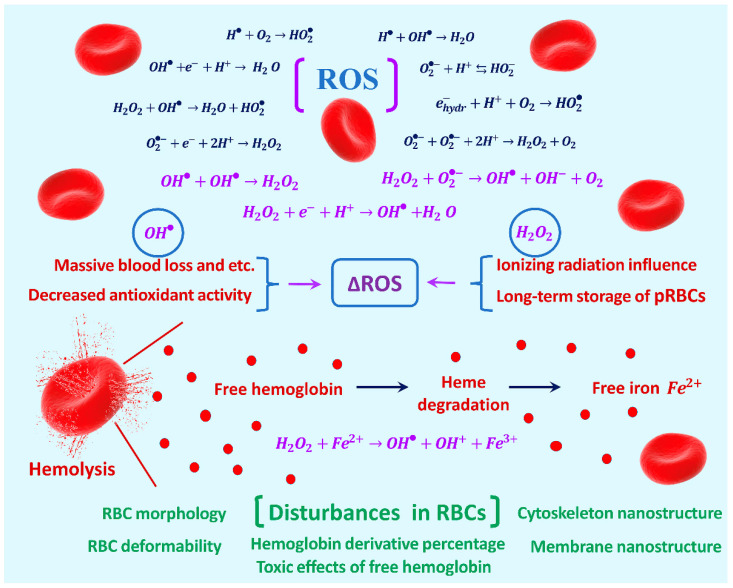
Patterns of chemical reactions between ROS. Formation of additional ΔROS as a result of various influences on RBCs. Reactions involving $OH^{•}$ leading to the formation of $H_{2}O_{2}$ and vice versa are highlighted in purple. A special role is played by Fenton’s reaction of $H_{2}O_{2}$ interacting with free iron $Fe^{2+}$, which occurs during hemolysis of RBCs. As a result, additional disturbances occur in RBCs.

Various reactions involving ROS occur in the blood (Figure 1). The presence of $Fe^{2+}\;$ in hemoglobin is characteristic of RBCs during redox processes. The hemolysis of RBCs also produces free $Fe^{2+}$ as a result of heme degradation, leading to the initiation of the Fenton reaction [22,23]:

(1) $$Fe^{2+}+H_{2}O_{2}=>Fe^{3+}+OH^{-}+OH^{•}.$$

In this important reaction, the $H_{2}O_{2}$ molecules are converted into $OH^{•}$ free radicals, which are specific ROS. They can initiate the chain reaction of lipid peroxidation in RBC membranes. The increase in the membrane permeability caused by lipid peroxidation has serious consequences for cells.

**Figure 2:**
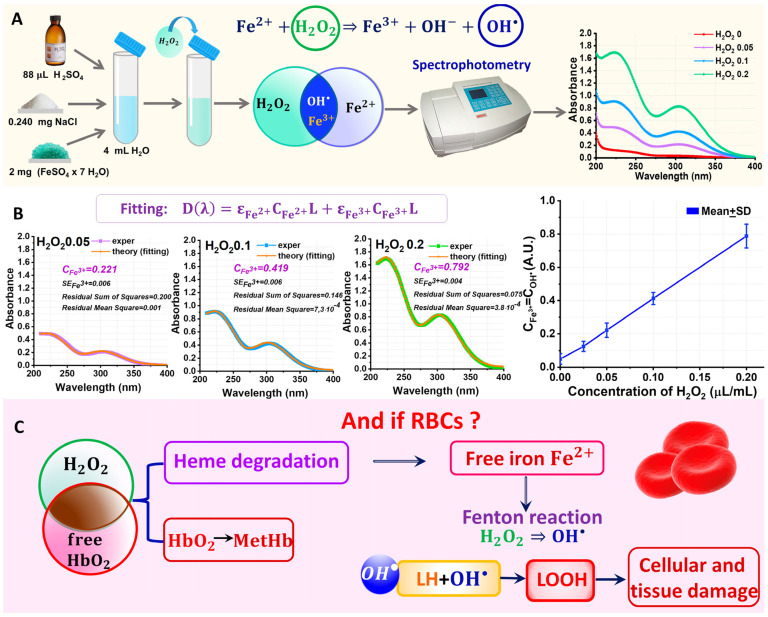
Quantitative study of $H_{2}O_{2}$ to $OH^{•}$ conversion due to the Fenton reaction in in vitro experiment. (**A**) Experimental design with $H_{2}O_{2}$ and $Fe^{2+}$. (**B**) On the left are the fitting results (red lines) of the experimental absorption spectra (green, cyan, and violet symbols) for the samples $H_{2}O_{2}0.2$, $H_{2}O_{2}0.4$, and $H_{2}O_{2}0.8$. The calculated concentrations $C_{Fe^{3+}}$, the SE of fitting, and residuals are indicated on each graph. The dependence $C_{Fe^{3+}\;}\left(C_{H_{2}O_{2}}\right)$, which corresponds to the dependence $C_{OH^{}}\left(C_{H_{2}O_{2}}\right)$, is shown on the right; the data are presented as mean ± SD. (**C**) Consequences of the interaction of $H_{2}O_{2}$ molecules and RBCs include MetHb formation, heme degradation production of free $Fe^{2+}$, generation of $OH^{•}$ due to the Fenton reaction, initiation of a chain LPO reaction in membranes, and damage to cells and tissues.

In our experiments, the dependence of $C_{OH^{•}\;}$ on $\left(C_{H_{2}O_{2}}\right)$ was determined quantitatively. To determine the kinetics of $C_{OH^{•}\;}\left(t\right)$, we measured the kinetics $C_{Fe^{3+}}\;\left(t\right)$ according to Equation (1). The experiment was carried out according to the scheme shown in Figure 2A. The $H_{2}O_{2}$ solution at different concentrations was added to the working solution containing $FeSO_{4}$. To quantify the concentration of $\;Fe^{3+}$ in the solution at any time point, spectrophotometry and nonlinear curve fitting (NCF) methods were used. The technique is described in detail in Section 3: Materials and Methods.

Alterations to the absorption spectrum of the experimental sample after adding $H_{2}O_{2}$ was demonstrated in the experiments (Figure 2A,B). Thus, peaks appear in the absorption spectrum at λ_1_ = 224 nm and λ_2_ = 304 nm, which are typical for ions $Fe^{3+}$ [41]. With an increase in the concentration of $C_{H_{2}O_{2}}$, the peaks increase in amplitude. This indicates the production of $Fe^{3+}$ ions in the solution as a result of the Fenton reaction.

To quantify the unknown concentration $C_{Fe^{3+}}$, we used the nonlinear curve fitting method. For this, the optical spectrum $D_{l}{\left(\lambda_{l}\right)}_{exper}$ was approximated using the theoretical curve $D_{l}{\left(\lambda_{l}\right)}_{theor}$:

(2) $$D_{l}{\left(\lambda_{l}\right)}_{theory}=F\left(\varepsilon_{Fe^{3+}},\varepsilon_{Fe^{2+}},C_{Fe^{3+}},C_{Fe^{2+}}\right).$$

NCF is described in detail in the Materials and Methods. Figure 2B on the left shows the measured spectra $\;D_{l}{\left(\lambda_{l}\right)}_{exper}$ and the corresponding fitting curves $D_{l}{\left(\lambda_{l}\right)}_{theor}$ constructed for the parameters calculated by Equation (2). $C_{Fe^{3+}}\;$ is an important biophysical parameter since $C_{Fe^{3+}}=C_{OH^{•}}$. Thus, for the $H_{2}O_{2}0$ (control) concentration, $C_{Fe^{3+}}=0.05$ (SE=0.01); for $H_{2}O_{2}0.05$, $C_{Fe^{3+}}=0.22$ (SE=0.01); for $H_{2}O_{2}0.1,$ $C_{Fe^{3+}}=0.41$ (SE=0.01); and for $H_{2}O_{2}0.2$, $C_{Fe^{3+}}=0.79$. All measurements are in arbitrary units (A.U.).

**Figure 3:**
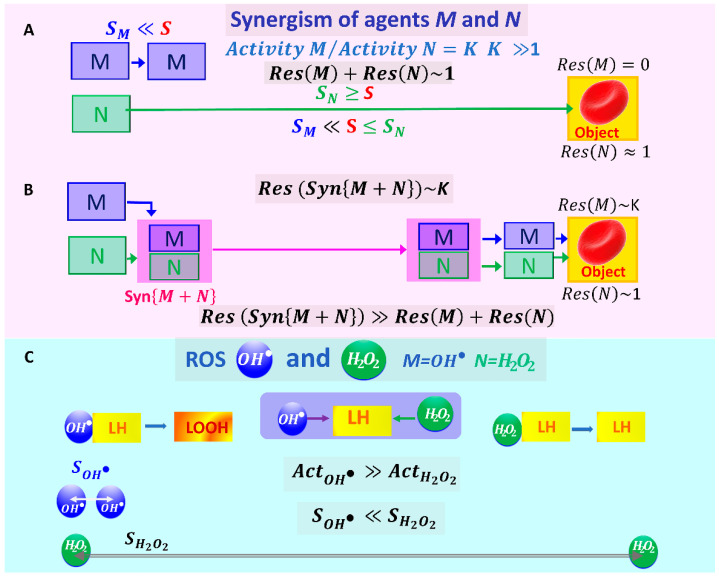
Schematic representation of the mechanochemical synergism effect of two factors M and N with very different levels of action on the RBC membrane $\;Act_{M}/Act_{N}=K$ and, accordingly, with a different typical diffusion path $S_{M}\ll S\leq S_{N}$. (**A**) Comparison of the results of the action of agents M and N on RBC without synergism. (**B**) Comparison of the results of the action of agents M and N on RBC with synergism. (**C**) Problem definition of mechanochemical synergism under the action of agents $M=OH^{•}$ and $\;N=H_{2}O_{2}$.

Figure 2B on the right shows the quantitative dependence of $C_{Fe^{3+}\;}(C_{H_{2}O_{2}})\;$ based on the subtraction of the control values. Thus, an increase in the concentration of $H_{2}O_{2}$ in the selected concentration range resulted in a corresponding linear increase in $C_{Fe^{3+}}$ with the proportionality coefficient β≈4 (A.U.) (Figure 3B).

Since, according to the Fenton reaction (Equation (1)), $C_{Fe^{3+}}=C_{OH^{•}}$, then the curve in Figure 2B corresponds to the kinetics of the change in $C_{OH^{•}}\;$ depending on $C_{H_{2}O_{2}}$:

(3) $$C_{OH^{•}}=\beta C_{H_{2}O_{2}}.$$

That is, the dependence $C_{OH^{•}}\left(C_{H_{2}O_{2}}\;\right)$ is also linear.

This reaction plays an important role in the functioning of RBCs. The interaction of $H_{2}O_{2}$ with the extracellular molecules of Hb resulting from RBC hemolysis (Figure 2C) ultimately leads to heme degradation and the appearance of free iron $Fe^{2+}$ in the RBC suspension (Figure 1 and Figure 2C) [22]. The Fenton reaction (Equation (1)) occurs in the RBC suspension. The resulting $OH^{•}$ free radicals are the main free radicals that induce the lipid peroxidation of the cell membrane. The lipid peroxidation of RBC membranes causes a disruption to the nanostructure of the membranes and their cytoskeleton, an increase in the membrane’s rigidity and permeability, as well as a disruption to the cell morphology up to their hemolysis (Figure 2C). As a result of the interaction of $H_{2}O_{2}$ with hemoglobin, the oxidation $Fe^{2+}\;\to \;Fe^{3+}$ can also occur, resulting in the conversion of $HbO_{2}$ to  MetHb, which is detrimental for the functioning of RBCs.

### 2.2. Mechanochemical Synergism of Two Factors in Their Effect on RBCs

Synergism is the interaction or cooperation of two factors to produce a combined effect trait which is greater than the sum of their individual effects.

In our experiments, we examined the following possible mechanochemical synergism (Figure 3A) in the suspension of RBCs.

Suppose the activity of the agent M on a biological object (BO) significantly exceeds the activity of the agent N on the same object:

(4) $$Act_{M}\gg Act_{N},\;Act_{N}=1,\;\;\;\;\frac{Act_{M}}{Act_{N}}=K,\;\;K\gg 1,\;\mathrm{for}\;\mathrm{example},\;K=10^{6}.$$

RBCs and their membranes can be considered BOs. The possible effects of two factors, M and N, are shown in Figure 3A,B. Due to the different activity of the agents, the typical lifetime τ and thus the typical diffusion path length of these agents, S, will differ significantly: $\tau_{N}\gg \tau_{M}$, $S_{N}\gg S_{M}$.

The probability that agent M is immediately next to a BO, which is at a distance $S\gg S_{M}$, is very small. Therefore, the result of agent M’s action will be 0 (Figure 3):$\;Res_{M}=0$.

The probability that agent N is immediately next to a BO is high if $S_{N}\geq S$, but its impact activity is small $,\;\;Res_{N}=1$. Then, the result (*Res*) of the total impact of agents M and N without their synergistic interaction will be:$\;Res\;\left(M\right)+Res\left(N\right)\sim 1$.

Agent N can cooperate with agent M, $Syn\left\{M+N\right\}$, that is, deliver it in some way to the BO (RBC membrane). In this case, the total result of the action will approach the value of $K=10^{6}$ (Equation (4)). Thus, the result of the action of factors M and N during their synergistic interaction will be many times greater than the sum of the results of the action of these agents individually:

(5) $$Res\left(Syn\left\{M+N\right\}\right)\gg Res\left(M\right)+Res\left(N\right)$$

### 2.3. Biophysical Basis for Identification of a Pair of ROS That May Be Involved in the Synergistic Process in Blood

As a result of various factors (decreased antioxidant activity, long-term storage of pRBCs, effects of ionizing radiation, massive blood loss, viruses, bacteria, etc.) on the blood, excited atoms and molecules, radicals, and ions appear. There is a swarm of ROS. The amount of ROS will be increased by the value of ΔROS (Figure 1). Excited molecules and radicals undergo all kinds of transformations. The molecules that burst out of the swarms can damage RBC membranes. This leads to changes in the structure of RBCs at various levels in terms of their properties and functions (Figure 1 and Figure 2C). Figure 1 shows some of the chemical reactions in which ROS are involved in in biological objects, especially in RBCs. Note that only ROS without active nitrogen forms and lipids are shown.

Depending on the environment, ROS have different spatio-temporal characteristics of interactions with each other. A short lifetime τ of corresponding radicals is usually typical of the excited state: superoxide oxygen anion radical $\tau_{O_{2}^{•-}}∼10^{-6}s,$ hydroperoxy radical $\tau_{HO_{2}^{•}}∼10^{-8}s,$ singlet oxygen $\tau_{O_{2}^{1}}∼10^{-6}s$, and hydroxyl radical $\tau_{\;OH^{•}}∼10^{-9}-10^{-6}s$ [4,42,43].

Therefore, the distances for their diffusion in a biological object are very short. This means that radicals and excited molecules are practically unable to escape from the swarm. That is why the radicals themselves can practically reach the membrane lipids, if the formation of ΔROS, for example, due to ionization, has taken place exactly in the lipid molecules or very close to them. 

In the ROS series, we distinguish two objects: hydroxyl radical $OH^{•}$ and hydrogen peroxide $H_{2}O_{2}$ (Figure 1 and Figure 3C). Reactions involving these ROS are highlighted in purple in Figure 1. The $OH^{•}$ radical is very active with respect to the lipid molecules of the RBC membranes. When it is located directly next to the lipid molecule *LH*, it is able to initiate a chain process of lipid oxidation, leading to the formation of lipid hydroperoxides LOOH. Accordingly, $OH^{•}$ is short-lived. It cannot travel long distances and therefore cannot damage molecules in the RBC membrane if the cell is far from the source of the radical. This is shown schematically in Figure 3C. The interaction of these radicals during their action on biological objects is also discussed in [44].

The activity of the $H_{2}O_{2}$ molecule is low, including a small effect on the RBC membrane lipid molecules. The $H_{2}O_{2}$ molecule is a long-lived ROS. Correspondingly, it can travel relatively long distances (Figure 3C).

(1) Estimates are presented for comparison. The oxidative effect of $OH^{•}$ is significantly higher than that $H_{2}O_{2}$: $\;A_{OH^{•}}\gg A_{H_{2}O_{2}}$ [4,41].

Relative activities with respect to lipids: $A_{H_{2}O_{2}}=1$.$\;A_{OH^{•}}=10^{9}$. Thus, $A_{OH^{•}}/A_{H_{2}O_{2}}=10^{9}$.

(2) The characteristic lifetimes of these two ROS differ significantly: $\tau_{H_{2}O_{2}}\;\sim 1-100\;s$, $\tau_{OH^{•}}=10^{-9}-10^{-6}s$ [4,43,44]. So, $\tau_{H_{2}O_{2}}\gg \tau_{OH^{•}}$.
(3) Correspondingly, the diffusion distances for these particles also vary considerably: $S_{H_{2}O_{2}}\gg S_{OH^{•}}$.

**Figure 4:**
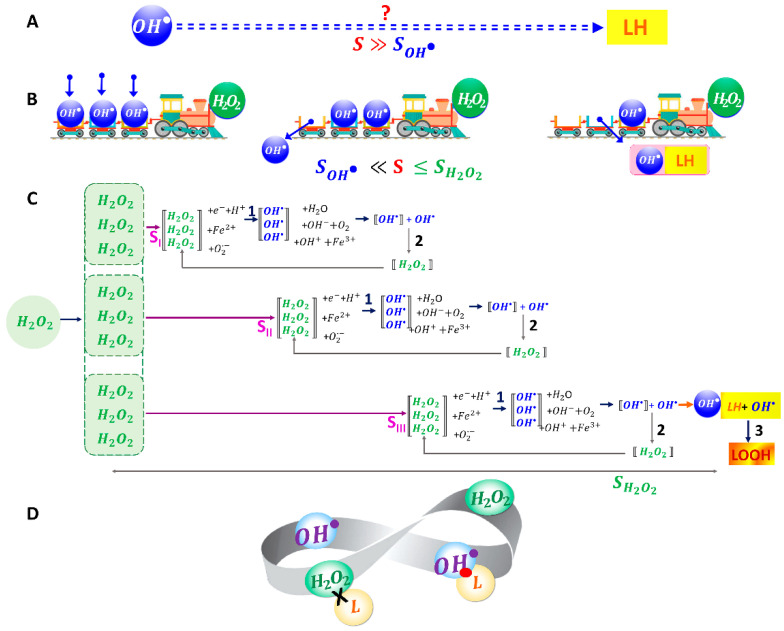
Mechanochemical synergism of $OH^{•}$ and $H_{2}O_{2}$ during action on the RBC membrane. (**A**) The problem of delivery of a highly active radical $OH^{•}\;$ to an object located at a distance exceeding its free path. (**B**) Schematic mechanistic representation of the assistance of $H_{2}O_{2}$ molecules along their path in the transport and release of $OH^{•}$. (**C**) Reactions of mutual transformation of the radicals $OH^{•}$ and $H_{2}O_{2}$ molecules. Examples are provided for 9$\;H_{2}O_{2}$ molecules. Three branches correspond to different distances $S_{I}$, $S_{II}$, and $S_{III}$, at which $H_{2}O_{2}$ interacted with other molecules and ions, resulting in the formation of $OH^{•}$ radicals at these distances (3 reaction options are shown)—stage 1. Radicals $OH^{•}$ can interact with each other at distances $S_{I}$, $S_{II}$, and $S_{III}$, resulting in the formation of new $H_{2}O_{2}$ molecules—stage 2, and can also interact with the adjacent membrane lipids *LH* of RBCs ($S_{III}$)—stage 3. (**D**) Figurative representation of $OH^{•}$ and $H_{2}O_{2}$ synergism in LPO.

Why did we choose $OH^{•}$ out of all the possible radicals? The fact is that $OH^{•}$ radical triggers a lipid peroxidation chain reaction (Figure 3C and Figure 4C).

### 2.4. Synergism of $OH^{•}$ and $H_{2}O_{2}$ When Acting on Biological RBC Membranes

This raises the question of whether $H_{2}O_{2}$ molecules can assist free radicals $OH^{•}$ in damaging lipids. In other words, can these ROS act synergistically when targeting the RBC membrane lipids? How can synergism be achieved in the specific case of $OH^{•}$ and $H_{2}O_{2}$?

Let us consider the mechanism of mechanochemical synergism according to the scheme shown in Figure 4C.

As noted above, the free radical $OH^{•}$ cannot move from its point of origin to the point where the target (RBC membrane) is located at a distance $S\gg S_{OH^{•}}\;$(Figure 4A). Let us also assume that $S_{OH^{•}}\;\ll S<S_{H_{2}O_{2}}$.

What could be the possible delivery route for highly active free radicals $OH^{•}$ using long-lived $H_{2}O_{2}$ molecules? The solution to this problem is schematically presented in Figure 4B using a mechanistic example. The $H_{2}O_{2}$ molecules play the role a vehicle for the delivery of $OH^{•}$. As it moves, the vehicle delivers free radicals $OH^{•}$ to various points, including directly to the RBC membranes. This ensures the impact of highly active free radicals $OH^{•}$ on RBC membrane lipids.

Obviously, this is only a schematic mechanistic representation of the mechanochemical synergism of the agents $H_{2}O_{2}$ and $OH^{•}$. In reality, the free radicals $OH^{•}$ and molecules $H_{2}O_{2}$ actively participate in many types of chemical processes (Figure 1). At the same time, there are several reactions in which they change into each other. From all chemical reactions shown in Figure 1, we have selected and included in the model only those reactions where $OH^{•}$ and $H_{2}O_{2}$ participate together (Figure 4C).

In solution, the $H_{2}O_{2}$ molecules participate in chemical reactions with $e^{-}+H^{+}$, $Fe^{2+}$, and $O_{2}^{•-}$, resulting in an increase in free radicals $OH^{•}$—stage 1 (Figure 4C). This happens as $H_{2}O_{2}$ diffuses in solution at a distance $S_{I}<S_{II}<S_{III}$.

In turn, hydroxyl radicals $OH^{•}$ can interact with each other to form the molecules $H_{2}O_{2}$; this process occurs rapidly in a short time and at a short distance—stage 2 (Figure 4C). Thus, the concentration of $H_{2}O_{2}$ molecules can partially recover in points $S_{I}$, $S_{II}$, and $S_{III}$. This feedback is shown by the arrows in Figure 4C. The synergism of chemical processes involving $OH^{•}$ and $\;H_{2}O_{2}$ can occur in solution as $H_{2}O_{2}$ molecules diffuse.

However, if $OH^{•}$ met the molecules of membrane lipids *LH* at a distance $S_{III}$, then it would also interact with them (Figure 4C, line III)—stage 3 (Figure 4C). This is a rapid process. As a result, at a distance of $S_{III}$, free radicals $OH^{•}$ will participate in two chemical processes, namely, their interaction with each other (with the reduction in $H_{2}O_{2}$ molecules) and their interaction with lipid molecules (without the reduction in $H_{2}O_{2}$ molecules)—corresponding to stages 2 and 3 (Figure 4C).

Figure 4D schematically shows the synergism of $OH^{•}$ and $H_{2}O_{2}$. Free radicals $OH^{•}$ rapidly initiate the peroxidation chain reaction. $H_{2}O_{2}$ molecules themselves cannot oxidize lipids but they can participate in the delivery of radicals to the membrane of biological objects. This schematic representation shows the interconversion of $OH^{•}$ and $\;H_{2}O_{2}$ in space and time.

This is the first level of the mechanochemical synergism of $OH^{•}$ and $H_{2}O_{2}$, namely, $H_{2}O_{2}$ molecules “deliver” free radicals $OH^{•}$ to cell membranes.

In the work, a kinetic modeling of synergism based on biophysical principles has been proposed. The synergism of free radicals $OH^{•}$ and $H_{2}O_{2}\;$ molecules allows us to organize a positive feedback system in RBC suspension. In this case, the effective distance of $OH^{•}$ action on the RBC membrane will increase significantly due to synergism, and the diffusion distance of $H_{2}O_{2}$ molecules will also increase.

Simultaneously with the first level, the second level of synergism also occurs, namely, free radicals $OH^{•}$ interacting with each other, generating $H_{2}O_{2}$ molecules, thus partially replenishing their loss (Figure 4C). The synergism of free radicals $OH^{•}$ and $H_{2}O_{2}$ molecules allows for organizing a positive feedback system in RBC suspension. As more $H_{2}O_{2}\;$ molecules are consumed, more free radicals $OH^{•}$ are produced (Figure 4C, stage 1), and in turn, more $H_{2}O_{2}$ molecules are produced (Figure 4C, stage 2), which compensates for the losses in stage 1. The process becomes cyclic. In this case, the effective distance of $OH^{•}$ action on the RBC membrane will increase significantly due to synergism, and the diffusion distance of $H_{2}O_{2}$ molecules will also increase.

### 2.5. Kinetic Model of the Mechanochemical Synergism of $H_{2}O_{2}$ and $OH^{•}$ Effects on RBC Membrane: The Role of Synergism in the Initiation of RBC Membrane LPO

Let us consider the mathematical model of the synergism involving $H_{2}O_{2}$ and $OH^{•}$.

The first level of mechanochemical synergism. This synergism delivers $OH^{•}$ radicals to RBC membranes, where they initiate LPO, as shown schematically in Figure 3 and Figure 4.

The molecule $H_{2}O_{2}$ is involved in chemical processes, some of which result in the formation of $OH^{•}$ (Figure 1):

(6) $$H_{2}O_{2}+e^{-}+H^{+}\to OH^{•}+H_{2}O,\;H_{2}O_{2}+Fe^{2+}\to OH^{•}+OH^{+}+Fe^{3+},\;H_{2}O_{2}+O_{2}^{•-}\to OH^{•}+OH^{-}+O_{2}.$$

As a result of these reactions, $C_{H_{2}O_{2}}\left(t\right)$ will decrease over time. Let us derive a differential equation to describe this process.

Let us designate:

$x\left(t\right)$—concentration of $OH^{•}$ radicals depending on time, $C_{OH^{•}}\left(t\right)$;

$y\left(t\right)$—concentration of $H_{2}O_{2}$ molecules depending on time, $C_{H_{2}O_{2}}\left(t\right)$;

z—concentration of all components with which $H_{2}O_{2}$ molecules interact.

In the equations, consider a time t that which is much longer than the typical lifetime of the radicals $OH^{•}$, but comparable to the lifetime of $H_{2}O_{2}$ molecules.

A differential equation of the change in y, stage 1 (Figure 4C):

(7) $$\frac{dy}{dt}=-kzy.$$

Initial condition: suppose at some point that $t=0,\;y=y_{0}$. Suppose that:

(8) $$y_{0}=100,$$

and k is the rate constant of the reaction.

Note that in the calculations, all concentrations of chemical components are presented as arbitrary units (A.U.), which are not specified, including in the graphs.

Then, we obtain:

(9) $$y=y_{0}e^{-kzt}.$$

Suppose that:

(10) k=1.

**Figure 5:**
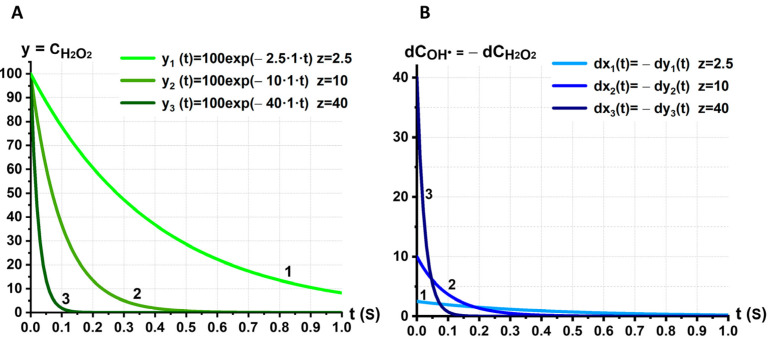
Kinetics of ROS reduction in the RBC system over time for different concentrations z=2.5, z=10, z=40. (**A**) The dependence $C_{H_{2}O_{2}}\left(t\right)$. (**B**) The dependence $dC_{OH^{•}}\left(t\right)=-dC_{H_{2}O_{2}}\left(t\right)$.

The graph of $y\left(t\right)$ is shown in Figure 5A. The kinetics of the concentration change over time; $C_{H_{2}O_{2}}\left(t\right)$ is shown for the three concentrations of values z with which $H_{2}O_{2}$ interacts: z=2.5, z=10, z=40. As the concentration z increases, the typical time of the reduction in y decreases.

The change in the value of y occurs due to the conversion of $H_{2}O_{2}$ into $OH^{•}$ (Figure 4C). Thus, the loss $dy\left(t\right)$ is equal to the increase in hydroxyl radicals $dx\left(t\right)$ over the time interval dt:

(11) dx=−dy=kzydt.

As a result, $OH^{•}$ radicals will be able to appear in the RBC suspension during the time $\tau_{OH^{•}syn}$ when they are produced under synergism by long-lived $H_{2}O_{2}$ molecules; that is, during the time which is much longer than their own lifetime: $\tau_{OH^{•}syn}\gg \tau_{OH^{•}}$.

The second level of mechanochemical synergism. There is *a* positive feedback system between $H_{2}O_{2}$ and $OH^{•}$ and, therefore, a partial recovery of consumed $H_{2}O_{2}$ molecules.

For different times, the values of $dx\left(t\right)$ will be different, because the $H_{2}O_{2}$ concentration decreases with time according to Equation (9). Consider a small interval where dy is not significant, so that dy/y  is no more than 10%.

For the parameters in Equation (9), the time interval is $dt=10^{-2}\;s$.

Let us take the concentration of radicals $OH^{•}$ formed during this time interval $dt=10^{-2}\;s$ as their initial concentration at t=0 for stages 2 and 3:

(12) $$x\left(t=0\right)=x_{0}.$$

These radicals will participate in further conversions, causing their reduction $x\left(t\right).$ Two main processes will be responsible for their reduction:

(1) Interaction of $OH^{•}$ with each other, stage 2 (Figure 4C):
  $$OH^{•}+OH^{•}\to H_{2}O_{2}$$
(2) Interaction with membrane lipids *LH*, stage 3:
  $$LH+OH^{•}\to \;L^{•}+H_{2}O$$

The interaction of $OH^{•}$ with each other (stage 2, Figure 4C) is described by the differential equation:

(13) $$\frac{dx}{dt}=-ax^{2},\;$$

where a is the rate constant of this chemical reaction.

Initial condition:

(14) $$x\left(t=0\right)=x_{0}.$$

When $OH^{•}$(*x*) interacts with lipids *LH* (stage 3, Figure 4C), there is a decrease in the lipid concentration $L\left(t\right)$ and $x\left(t\right)$. This process is described by the differential equation:

(15) $$\frac{dL}{dt}=-bLx,\;$$

accordingly, the change in $x\left(t\right)$ in this case will be:

(16) $$\frac{dx}{dt}=-bLx,\;$$

where b is the reaction rate constant.

The kinetics of the change in $x\left(t\right)$ during the simultaneous occurrence of both processes during stages 2 and 3 (Figure 4C) is described by a differential equation:

(17) $$\frac{dx}{dt}=-ax^{2}-bLx.\;$$

Initial condition:

(18) $$\;\;x\left(t=0\right)=x_{0},\;L\left(t=0\right)=L_{0}.$$

Then, the solution is:

(19) $$x\left(t\right)=\frac{bL}{-a+e^{bL\left(t+\;\frac{ln(\left(bL+ax_{0}\right)/x_{0})}{bL}\right)}}.$$

**Figure 6:**
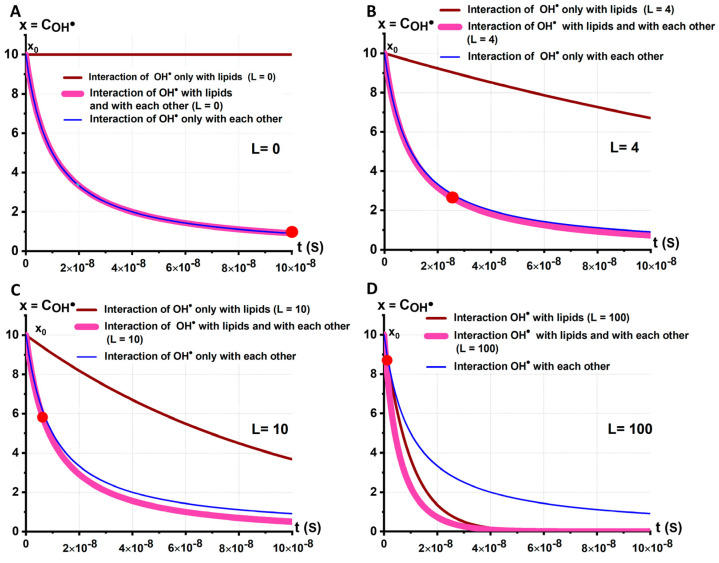
Graphs of the change in $x\left(t\right)$ (Equation (19)) at different lipid concentrations. (**A**) L=0, $q_{1}$. (**B**) L=4, $q_{2}$. (**C**) L=10, $q_{3}$. (**D**) L=100, $q_{4}$. Replenishment coefficients: $q_{1}>q_{2}>q_{3}>q_{4}$. Coefficient $a=10^{6}$, $k=10^{7}$.

Figure 6 shows the kinetic $C_{OH^{•}}\left(t\right)$ for different lipid concentrations  L. Moreover, for each graph corresponding to a given concentration L, three curves are presented for three cases: only stage 3 (brown line), only stage 2 (blue line), and both stages (2 + 3) simultaneously (purple line).

If L=0, there would be no change in $OH^{•}$ if it will be only stage 3 (Figure 4C). In this case, $x\left(t\right)$ decreases only due to the interaction of $OH^{•}$ among themselves (blue and purple lines coincide) (Figure 6A). Additionally, this would lead to the partial recovery of the reduced $H_{2}O_{2}$ with the replenishment coefficient $q_{1}$.

If L=10, up to the time $t=6\cdot 10^{-9}s$, the loss of $x\left(t\right)$ would be determined mainly by the interaction of x among themselves. This part of the radicals can turn into $H_{2}O_{2}$ and thereby partially replenish the loss of these molecules; replenishment coefficient $q_{2}$.

If L=100, the interaction of x with L (stage 3) plays a major role and stage 2 hardly occurs. In this case, the replenishment coefficient $q_{4}$ is small. Among replenishment coefficients, the following inequality is satisfied: $q_{1}>q_{2}>q_{3}>q_{4}$.

The possibility of replenishing the loss of molecules $H_{2}O_{2}$ due to the interaction of $OH^{•}$ with each other leads to the decrease in the effective reaction rate constant k in Equations (7)–(11). What does this lead to?

**Figure 7:**
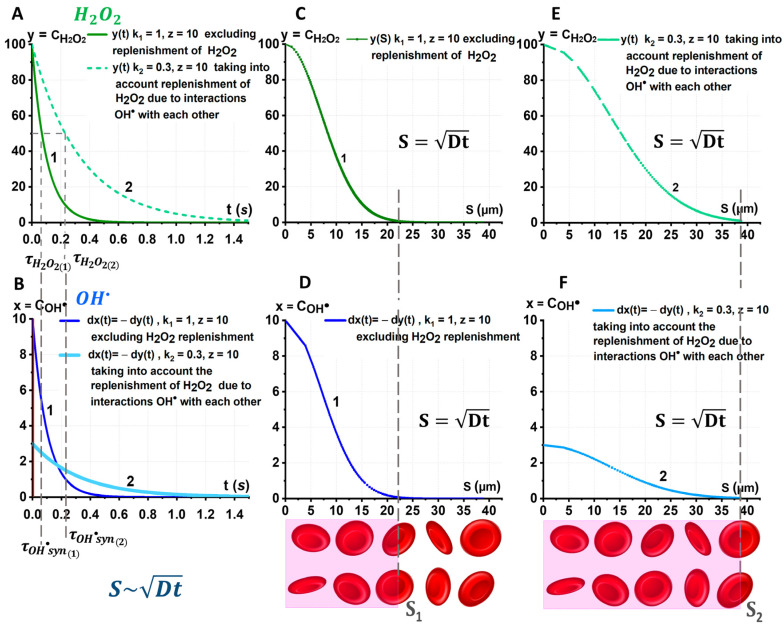
Effect of mechanochemical synergism on distribution of $OH^{•}$ and $H_{2}O_{2}$. (**A**) $y\left(t\right)=C_{H_{2}O_{2}}\left(t\right)$ without (1) and with consideration of the replenishment of $H_{2}O_{2}$ (2). (**B**) Correspondingly, $x\left(t\right)=C_{OH^{•}}\;\left(t\right)\;$. (**C**,**D**) Spatial change $y\left(S\right)=C_{H_{2}O_{2}}\left(S\right)$ and correspondingly $x\left(S\right)=C_{OH^{•}}\;\left(S\right)$ without replenishment of y, k=1 (only first-level synergism). (**E**,**F**) Spatial change $y\left(S\right)=C_{H_{2}O_{2}}\left(S\right)$ and accordingly $x\left(S\right)=C_{OH^{•}}\;\left(S\right)$, taking into consideration the restoration of y, k=0.3 (first and second levels of synergism). Below, for comparison, there is an illustration of the diffusion distance for $H_{2}O_{2}$ and $OH^{•}$ under synergism without (**C**,**D**) and with (**E**,**F**) the corresponding reduction in y; the thicknesses of the RBC layer that can be affected by $OH^{•}$ are highlighted in pink: $S_{1}$ considering only first-level synergism, $S_{2}$ considering synergism of first and second levels. The diffusion distance $S=\sqrt{Dt}$.

Figure 7A,B shows the relations $C_{H_{2}O_{2}}\left(t\right)$ and $C_{OH^{•}}\;\left(t\right)$ for various k. Coefficient $k_{1}$ corresponds to the process without the recovery of $H_{2}O_{2}\;$ due to stage 2; coefficient $k_{2}$ corresponds to process with the recovery of $H_{2}O_{2}\;$ due to stage 2. In graphs 1 and 2 (Figure 7A), the lifetimes $\tau_{H_{2}O_{2\left(1\right)}}$ and $\tau_{H_{2}O_{2\left(2\right)}}$ are indicated. Lifetime $\tau_{H_{2}O_{2}}$ is the time for the $H_{2}O_{2}\;$ concentration to halve. Correspondingly, the lifetimes $\tau_{OH^{•}syn_{\left(1\right)}}$ and $\tau_{OH^{•}\;syn_{\left(2\right)}}$, showing how long they can be generated during the synergetic process, are indicated in Figure 7B.

According to the model, $\tau_{OH^{•}syn{\;}_{\left(2\right)}}>\tau_{OH^{•}syn_{\left(1\right)}}$ (Figure 7A,B). The time $\tau_{H_{2}O_{2\left(2\right)}}$ is the effective lifetime, since it was obtained by taking into account the effect of the replenishment of $H_{2}O_{2}$ due to the interaction of hydroxyl radicals. This will lead to an increase in the duration of $OH^{•}$ generation. Figure 7B shows a red line near t=0 corresponding to a decrease in hydroxyl radicals over time in the absence of first-level synergism between $OH^{•}$ and $H_{2}O_{2}$. As already mentioned, the lifetime of the radicals $OH^{•}$ is very short: $\;\tau_{OH^{•}}\;\ll \tau_{OH^{•}syn_{\left(1\right)}}$.

ROS interaction occurs along their diffusion distances (Figure 7C–F). The diffusion distance can be estimated by the formula $S\sim \sqrt{Dt}$ [45]. For the radicals $OH^{•}$, it is very small. $H_{2}O_{2}$ plays the main role in the diffusion processes with ROS synergism: $S\sim \sqrt{D_{H_{2}O_{2}}\tau_{H_{2}O_{2}}}$, where $D_{H_{2}O_{2}}$ is the diffusion coefficient of $H_{2}O_{2}$ molecules. Considering that in water $D_{H_{2}O_{2}}∼10^{-5}\;{\mathrm{cm}}^{2}/s$ [45,46], we will obtain that the molecule $H_{2}O_{2}$ diffuses in RBC suspension over a distance of tens of microns in 1 s.

Knowing the dependences $y\left(t\right)$ and $x\left(t\right)$, and also considering that the distance over which the particle travels due to diffusion during time t can be estimated from the equation $S\sim \sqrt{Dt}$, we obtain the dependences $y\left(S\right)$ and $x\left(S\right)$. These dependencies are shown in Figure 7C–F.

The increase in the effective lifetime $\tau_{H_{2}O_{2}}$ due to the second level synergism of $OH^{•}$ and $H_{2}O_{2}$ leads to an increase in the diffusion distance.

Under the conditions of ROS synergism, the $H_{2}O_{2}$ molecules (Figure 7E, green curve) will be able to diffuse and consequently generate $OH^{•}$ (Figure 7F, blue curve) over longer distances $S_{2}$ considering the recovery of y than $S_{1}$ without the recovery (Figure 7C,D). Thereby, $OH^{•}$ radicals will cover a larger area of membrane lipids. For comparison, Figure 7 below shows the illustration of the diffusion distance for $H_{2}O_{2}$ and $OH^{•}$ under synergism for two cases: without (C, D) and with (E, F) the recovery of y*,* respectively. The thickness of the RBC layer, which can be affected by free radicals $OH^{•}$, $S_{2}>S_{1}\;$(highlighted in pink).

## 3. Materials and Methods

### 3.1. Fricke System as a Model for Quantitative Study of $H_{2}O_{2}$ to $OH^{•}$ Conversion Due to the Fenton Reaction in In Vitro Experiment

#### 3.1.1. Preparation of the Working Solution

Fricke’s solution was used as the working solution [41]. In the following, we will refer to this solution as solution A. For its preparation, 2 mg of $FeSO_{4}\cdot 7H_{2}O$ (Sigma-Aldrich, Saint Louis, MO, USA) and 0.240 mg of NaCl were weighted and then added to 4 mL of $H_{2}O$. To the resulting solution, 88 µL of $H_{2}SO_{4}$ 99.999% (Sigma-Aldrich, Saint Louis, MO, USA) were added. Thus, solution A was prepared. Next, different volumes of hydrogen peroxide $(H_{2}O_{2}$ 3%) (0, 0.1, 0.2, 0.4, and 0.8 µL) were added to solution A. Thus, solution B was prepared (Figure 2A). In this case, the Fenton reaction took place. The $Fe^{2+}$ ions were converted into $Fe^{3+}$ ions by oxidation, resulting in free radical $OH^{•}$ generation. This process is typical of RBCs.

#### 3.1.2. Spectrophotometry for Quantitative Analysis of Fenton Reaction Results

To determine the concentration of $OH^{•}$ in the studied solutions, we used the spectrophotometric method to measure the absorption spectrum of solution B. The optical absorption spectra of solution B were measured using a Unico 2800 digital spectrophotometer (United Products & Instruments, Dayton, FL, USA) (Figure 2A). The experimental spectra $D{\left(\lambda \right)}_{exper}$ were measured in the wavelength range of λ=200−400 nm with a step of 1 nm. The experimental spectra are shown in Figure 2A,B.

The maximum characteristic of $Fe^{3+}$ ions was observed at the wavelength λ=304 nm. We observed changes in the spectrum of solution B, namely, the absorption maximum amplitude increased with the rising concentration of $H_{2}O_{2}$. This indicated the conversion of $Fe^{2+}$ to $Fe^{3+}$. We need to determine the concentration of ${\mathrm{Fe}}^{3+}$ according to the chemical reaction (Equation (1)). Due to the fact that $C_{OH^{•}}=C_{Fe^{3+}}$, according to the Fenton reaction, we estimated the dynamics of $C_{OH^{•}}\;$ changes by analyzing the spectrum of solution B.

The working solution B with different concentrations of hydrogen peroxide was designated as $H_{2}O_{2}0$, $H_{2}O_{2}0.025$, $H_{2}O_{2}0.05$, $H_{2}O_{2}0.1$, and $\;H_{2}O_{2}0.2$ for $C_{H_{2}O_{2}}=0,\;0.025,\;0.05,\;0.1,\;\mathrm{and}\;0.2$ µL/mL, respectively.

#### 3.1.3. Nonlinear Curve Fitting of Optical Spectra for Determination of the Unknown Concentration $C_{OH^{•}}$

To determine the unknown concentration $C_{OH^{•}}$, it was necessary to perform nonlinear curve fitting of the wavelength dependences of the optical densities $\;D_{l}{\left(\lambda_{l}\right)}_{exper\;}$ obtained experimentally by spectrophotometry, where l   is the number of wavelengths and $\lambda_{l}$ is the set of the wavelengths.

We used software of Origin Pro 2019 (OriginLab Corporation, Northampton, MA, USA) for NCF of the experimental data. We used the absorption law function as the theoretical function for NCF:

(20) $$D_{l}{\left(\lambda_{l}\right)}_{theor}=\;\varepsilon_{Fe^{2+}}C_{Fe^{2+}}L+\varepsilon_{Fe^{3+}}C_{Fe^{3+}}L.$$

Equation (20) was created based on biophysical principles [47]. The fitting curve was chosen on the basis of the biophysical law of light absorption, the Bouguer–Beer–Lambert law, considering that absorption in a solution containing two ions ($Fe^{2+}$ ions, $Fe^{3+}$ ions) $D_{l}{\left(\lambda_{l}\right)}_{theor}$ is a nonlinear function. Here, $\varepsilon_{Fe^{2+}}\left(\lambda_{l}\right)$ and $\varepsilon_{Fe^{3+}}\left(\lambda_{l}\right)$ are molar absorptivity coefficients at given wavelengths $\lambda_{l}$. Molar absorptivity coefficients are the nonlinear functions of $\lambda_{l}$. We obtained these values based on the data of [41]. Thus, in the model, these parameters and layer thickness L=1 cm can be considered as the known parameters. To determine concentrations of $C_{Fe^{2+}},\;C_{Fe^{3+}}$ we made NCF in the whole range of experimental data 200–400 nm.

The unknown parameters in Equation (20) are the concentrations $C_{Fe^{2+}},\;C_{Fe^{3+}}$.

In Equation (20), instead of the theoretical massive $D_{l}{\left(\lambda_{l}\right)}_{theor}$, we used the experimental optical density data $D_{l}{\left(\lambda_{l}\right)}_{exper}$. The calculation of the fitted values in NCF is an iterative procedure performed using the Levenberg–Marquardt algorithm. Iteration to adjust parameter values continues to bring the theoretical curve with calculated model parameters closer to the experimental data. A number of studies have considered that the R-squared is not the most significant factor in non-linear analysis [48]. In our study, we reported the fitted value, standard errors, and the residuals of the fitting in the legend (in Figure 2B). The model parameters which were obtained from NCF are used to plot the theoretical function according to Equation (20). The $C_{Fe^{3+}}$ was obtained by fitting. Since $C_{OH^{•}}=C_{Fe^{3+}}$ as a result of the Fenton reaction, we actually obtained the $OH^{•}$ concentration for different $C_{H_{2}O_{2}}$.

### 3.2. Statistical Analysis

The data were analyzed using software of Origin Pro 2019. Optical spectra were measured five times for a given concentration of $H_{2}O_{2}$, and the mean concentrations of $\;Fe^{2+}$ ions, $Fe^{3+}$ ions, and $H_{2}O_{2}$ molecules were calculated by NCF. The experiments were performed 15 times for each $C_{H_{2}O_{2}}$. For each sample, the mean and standard error of $C_{Fe^{3+}}$ were calculated. These data are reported in the Results and Discussion section. All data in Figure 2B on the right are presented as the mean ± SD.

Thus, in the study, the fitted value, standard error of the fitting, and the residuals are reported as statistical parameters. The mean, SE, and SD of the sample parameters were also calculated.

### 3.3. Kinetic Model of the Synergistic Interaction between $H_{2}O_{2}$ and $OH^{•}$

Mathematical modeling was used to study the kinetics of change in the concentrations of $\;H_{2}O_{2}$ and $OH^{•}$. Based on the law of mass action, ordinary differential equations describing the kinetics of chemical reactions were recorded. The kinetic equations were solved using the method of the separation of variables, considering the initial conditions.

The basic assumptions of the kinetic model are as follows.

(1) We consider chemical processes of the conversion of $H_{2}O_{2}$ into $OH^{•}$ by interaction with other molecules and vice versa the conversion of $OH^{•}$ into $H_{2}O_{2}$.
(2) The second assumption is based on the significant difference in the lifetimes of the free radical $OH^{•}$ and the molecule $H_{2}O_{2}$. This allows the processes of decreasing/increasing concentrations of these ROS to be separated in time and described by individual differential equations.

As a result, the calculated kinetic curves $C_{H_{2}O_{2}}\left(t\right)$ and $C_{OH^{•}}\;\left(t\right)$ take into account the mutual transformation of $H_{2}O_{2}$ and $OH^{•}$. The interaction of $OH^{•}$ with RBC membrane lipid molecules is also considered.

## 4. Conclusions

Red blood cells are continuously exposed to both endogenous and exogenous sources of reactive oxygen species in the circulation. To minimize the effects of these ROS, RBCs possess an extensive antioxidant system. An imbalance between excessive ROS production and antioxidant defense results in oxidative stress. The study of the mechanisms of the occurrence of an excess number of ROS and their amplification during the functioning of the circulatory system remains the subject of intensive scientific research. This is due to the fact that oxidative stress plays a significant role in the damage of erythrocyte membranes, cell morphology, and deformability. The mechanisms of the processes involved in the appearance and development of these pathologies are still not fully understood. This is currently the subject of research and scientific discussion. ROS are characterized by a strong interrelation as they undergo chemical transformations. The same ROS can be both a consequence and a cause of chemical transformations of others. Such an overlapping of events between a large number of ROS makes the study and the interpretation of the results very difficult. In this work, based on the biophysical principles, a kinetic model of the mechanochemical synergism of the action of free radicals $OH^{•}$ and molecules $H_{2}O_{2}\;$ on the initiation of lipid peroxidation of RBC membranes is discussed.

The effectiveness of the impact of these ROS on RBC membranes under synergistic conditions has been shown to be many times higher than in the case of an exposure to the same factors individually. As a result of this ROS synergism, the efficiency of LPO in RBC membranes is greatly enhanced. A further study of the possible mechanisms of ROS synergism in blood will help to clarify the mechanisms of the destruction of bacteria and viruses in the blood, the damage of RBC membranes during radiotherapy and the long-term storage of packed RBCs, as well as the effects of toxins. This study is also important for the development of new methods to protect RBCs from excessive ROS.

## Data Availability

The datasets used and analyzed during the current study are available from the corresponding authors upon request.
